# Cluster dispersal shapes microbial diversity during community assembly

**DOI:** 10.1371/journal.pcbi.1013918

**Published:** 2026-02-02

**Authors:** Loïc Marrec, Sonja Lehtinen

**Affiliations:** 1 Département de Biologie Computationnelle, Université de Lausanne, Lausanne, Switzerland; 2 Swiss Institute of Bioinformatics, Lausanne, Switzerland; Abdus Salam International Centre for Theoretical Physics, ITALY

## Abstract

Identifying the drivers of diversity remains a central challenge in microbial ecology. In microbiota, within-community diversity is often linked to host health, which makes it all the more important to understand. Since many communities assemble de novo, microbial dispersal plays a critical role in shaping community structure during the early stages of assembly. While theoretical models typically assume microbes disperse individually, this overlooks cases where microbes disperse in clusters, such as, for example, during host feeding. Here, we investigate how cluster dispersal impacts species richness, between-community dissimilarity, and species abundance in the initial steps of microbial community assembly. We developed a model in which microbes disperse from a pool into communities as clusters and then replicate locally. Using both analytical and numerical approaches, we show that cluster dispersal promotes community homogenization by increasing within-community richness and reducing dissimilarity across communities, even at low dispersal rates. Moreover, it modulates the influence of local selection on microbial community assembly and, consequently, on species abundance. Our results demonstrate that cluster dispersal has distinct effects from simply increasing the dispersal rate. This work reveals new evidence for the role of cluster dispersal in the early dynamics of microbial community assembly.

## Author summary

Microbial communities, such as those living in the gut, play important roles in host health, yet we still do not fully understand how their diversity is established. In many cases, these communities assemble from scratch, making the way microbes move and colonize new habitats, known as dispersal, crucial for shaping community structure. While most models assume microbes disperse individually, in reality they can travel in clusters, for example, during feeding events. In this study, we explore how cluster dispersal affects the formation and diversity of microbial communities. Using a combination of mathematical modeling and computer simulations, we show that when microbes disperse in clusters, communities become more similar to each other, with higher species richness within each community. Cluster dispersal also changes how local environmental conditions influence which microbes thrive, altering patterns of species abundance. These effects are not simply explained by a higher dispersal rate, highlighting the unique impact of clustered movement.

## Introduction

Dispersal is a key driver of microbial community assembly [1]. Many microbial communities form from scratch when new microenvironments emerge. For example, many organisms do not inherit their parents’ microbiota and are therefore born with germ-free microbiota, which become populated after birth [e.g., *Caenorhabditis elegans* [2], *Drosophila melanogaster* [3]. The early dispersing microbes are crucial, as they shape community composition, metabolic activity, and the development of complex ecosystems and biogeochemical processes [4]. Thus, community assembly is a potentially critical phase in the development of microbial diversity.

In the context of host-associated microbiota, within-community microbial diversity is often linked to host health. However, the nature of this relationship varies depending on the body site. For instance, a healthy vaginal microbiota is typically characterized by low species richness [5]. Its composition becomes especially crucial during pregnancy, where it serves as a barrier against infections that could threaten both maternal and fetal health [5]. Conversely, in the gut, reduced microbial diversity is frequently associated with a range of adverse health outcomes [6], including inflammatory, metabolic, and immune-related disorders. Interventions aimed at increasing gut microbiota diversity, such as the use of prebiotics, probiotics, or fecal microbiota transplantation, have shown therapeutic potential in restoring microbial diversity and improving host health [7,8].

Microbial diversity encompasses more than just within-community variation. Microbial diversity also includes substantial differences between communities, often referred to as between-community dissimilarity (or *β*-diversity). Microbial communities can vary dramatically in composition and abundance across individuals, even among hosts of the same species [9–11]. Notably, even genetically identical individuals, such as monozygotic twins, often harbor markedly different microbiomes, highlighting the role of non-genetic influences in shaping microbial ecosystems [12,13].

Dispersal plays a key role in within- and between-community diversity. Limited dispersal increases between-community dissimilarity, while high dispersal rates homogenize community structures [14]. [15] experimentally confirmed this by assembling worm gut microbiotas with two species, demonstrating that richness increases with dispersal, whereas between-community dissimilarity decreases. They quantified this

transition using the bimodality coefficient [16], a summary statistic describing abundance fluctuation distributions [17], which is the distribution of abundances across communities that are replicates of a same assembly processes.

Building on [15]’s work, [18] developed a community assembly model to derive analytical predictions for the bimodality coefficient, refining assessments of dispersal’s role in richness and between-community diversity. They also introduced mean relative abundance, i.e., the extent to which the community is dominated by a single species, as an additional metric for comparing microbial traits across species.

Like many theoretical models (e.g., [19]), [18] assumed that microbes disperse individually. However, in host-associated communities, microbes are often ingested in clusters. For example, humans regularly ingest multiple microbes through food and water. Thus, a single meal often introduces a mix of multiple microbes into the human gut. Experimental setups can also create conditions that likely involve cluster dispersal: for instance, [20] experimentally showed that stochastic colonization leads to alternative stable states by measuring microbial establishment probabilities in the fruit fly *D. melanogaster* gut using inoculum doses from 10^1^ to 10^8^ CFUs. Similarly, [21] showed that colonization outcomes are shaped by both stochasticity and context-dependent interactions by feeding germ-free flies a 5 × 10^6^ CFU inoculum of bacterial combinations. These examples highlight that both natural feeding events and experimental designs can generate cluster dispersal. To date, it remains unclear how cluster dispersal impacts the assembly dynamics of microbial communities.

In this study, we investigate how cluster dispersal influences richness and between-community dissimilarity during the early stages of assembly. We develop a model in which two microbial species disperse from a pool into local communities, in which they replicate. Using both analytical and numerical approaches, we demonstrate that cluster dispersal tends to homogenize microbial communities and influences mean relative abundance in a non-monotonic way when combined with within-community selection. To assess the robustness of our predictions, we extend our model to multiple species and quantify *α*- and *β*-diversity. Overall, our work highlights the role of cluster dispersal in the early dynamics of microbial community assembly.

## Model and methods

### Microbial community assembly model

**Fig 1:**
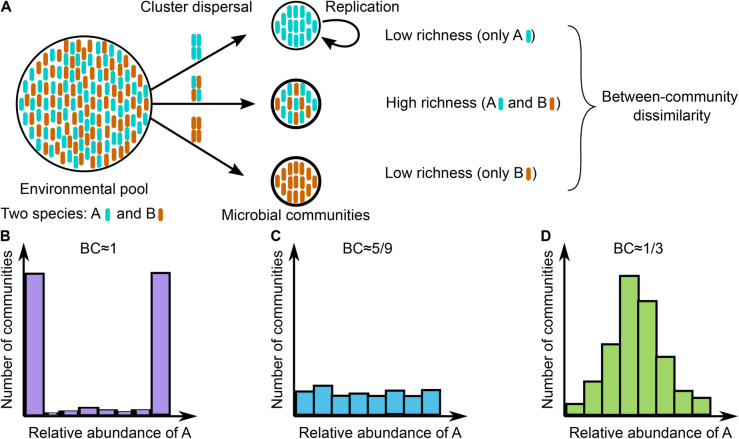
Sketch of the microbial community assembly model. As shown in Panel **A**, the environmental pool contains two species, A (blue) and B (orange). Species A is present in the pool in abundance $p_{A}$, while species B is present in abundance 1 − $p_{A}$. Microbial clusters of size *n* disperse from the pool into local microbial communities at rate *c* and replicate within these communities at rates $r_{A}$ and $r_{B}$. Cluster composition and abundance are drawn from a binomial distribution $ℬ(n,p_{A})$. Once each community reaches its carrying capacity, we analyze the abundance fluctuation distribution, which quantifies the number of communities with a given composition and abundance (**B**, **C**, and **D**). We characterize this distribution using its bimodality coefficient (BC) and mean relative abundance.

We build a model to represent the early stages of microbial community assembly, starting from initially microbe-free communities. This model, shown in Fig 1A, includes a microbial pool consisting of two species, A and B, present in abundances $p_{A}$ and 1 − $p_{A}$, respectively. Microbial clusters of size *n*, where *n* is an integer between 1 and *K*, disperse from the pool into local communities at a rate *c*. The composition and abundance of these clusters are drawn from a binomial distribution $ℬ(n,p_{A})$. The local communities are assumed to experience identical selective conditions, meaning that each species replicates at a fixed rate across all communities. This assumption is relevant to certain experimental setups involving clonal worms or flies [15,21,22].

Once introduced into a community, microbes replicate at rates that may depend on the species: $r_{A}$ for species A and $r_{B}$ for species B. Here, $N=N_{A}+N_{B}$ represents the total community size. We consider that the assembly process is complete when the community size reaches the carrying capacity (i.e., *N* = *K*).

To account for the limiting effects of carrying capacity *K* on community growth, the dispersal and replication rates are multiplied by a saturation term, namely (1 − N/K), which is derived from logistic growth dynamics [23]. This formulation reflects the reduced probability of successful replication or establishment as the community approaches its carrying capacity *K*, thereby capturing ecological effects such as competition for space.

Once community assembly is complete, defined here as the first time the total community size reaches the carrying capacity *K*, we quantify species richness and between-community dissimilarity (i.e., within- and between-community diversity, or *α*- and *β*-diversity, respectively) using the abundance fluctuation distribution [17]. The abundance fluctuation distribution describes the number of communities that contain a given abundance of A microbes (Fig 1B, 1C, and 1D). Because our model does not include turnover, the abundance fluctuation distribution becomes fixed once *N* = *K*. This assumption reflects our focus on the early stages of community assembly, where dynamics are driven by growth and dispersal rather than ongoing turnover. To characterize the abundance fluctuation distribution, we use the bimodality coefficient, denoted by BC, a summary statistic of probability distributions that ranges from 0 to 1 [16]. Values of BC exceeding 5/9 indicate bimodality, suggesting a low-dispersal assembly regime dominated by cell replication (Fig 1B). This regime typically leads to low richness but high between-community dissimilarity [15,18]. In contrast, BC values below 5/9 signify a unimodal distribution, characteristic of a high-dispersal regime where dispersal events dominate (Fig 1D), resulting in high richness and low between-community dissimilarity [15,18].

An additional descriptor of the abundance fluctuation distribution is its mean, which can be used to compare microbial traits across species and to detect processes such as selection [18].

Note that, while our model relies on several simplifying assumptions, we verify in S1 Text (Sect 8) how relaxing these assumptions affects community assembly and diversity patterns. In particular, we examine scenarios with dispersal without saturation (i.e., applying the logistic term (1−N/K) only to the replication rate), cell death, and random cluster sizes.

### Microbial community assembly simulation

We simulate the assembly of a microbial community using a Gillespie algorithm [24,25], which generates trajectories of stochastic dynamics based on known event rates. In our model, three types of events are considered: the replication of microbes from species A and species B


$$(N_{A},N_{B})\overset{r_{A}\left(1-\frac{N_{A}+N_{B}}{K}\right)N_{A}\;}{\to }(N_{A}+1,N_{B}),$$

and


$$(N_{A},N_{B})\overset{r_{B}\left(1-\frac{N_{A}+N_{B}}{K}\right)N_{B}\;}{\to }(N_{A},N_{B}+1),$$

where $[r_{A}]=[r_{B}]=1/\text{time}$, and the dispersal of clusters of size *n*


$$(N_{A},N_{B})\overset{c\left(1-\frac{N_{A}+N_{B}}{K}\right)\;}{\to }(N_{A}+n_{A},N_{B}+n-n_{A}),$$

where [c]=1/time, $n_{A}$ is the number of A microbes in a cluster, which is drawn from a binomial distribution $ℬ(n,p_{A})$, and $p_{A}$ is the abundance of species A in the microbial pool. For each event, the propensity function is indicated above the arrow. The simulation steps are as follows:

1. Initialization: The microbial community starts from $N_{A}=0$ and $N_{B}=0$ microbe.
2. Event selection: The next event to occur is chosen randomly proportionally to its probability. For example, the replication of a microbe of species A is chosen with probability $r_{A}N_{A}/(r_{A}N_{A}+r_{B}N_{B}+c)$.
3. Population size update: The population sizes $N_{A}$ and $N_{B}$ are updated according to the event selected in Step 2. For example, if the replication of a species A microbe is chosen, the population size of species A is updated by $N_{A}\to N_{A}+1$.
4. We return to Step 2 until the community size is equal to the carrying capacity ($N_{A}+N_{B}=K$).

Each stochastic realization of the above algorithm describes the assembly of a single microbial community. Thus, collecting several stochastic realizations is equivalent to simulating the microbial assembly of several communities.

## Results

### Cluster size and dispersal intensity determine the balance of processes driving community assembly

Distinguishing the contributions of dispersal and replication is a crucial first step toward understanding the patterns of diversity that emerge during community assembly. Our model conceptualizes community assembly as the result of two processes: within-community cell replication and cluster dispersal. These processes can occur at distinct rates, which determines their relative contributions to community assembly. The balance between these contributions naturally emerges from the comparison of their respective rates. [18] demonstrated that two distinct assembly regimes can be identified: a low-dispersal regime, where community assembly is predominantly driven by cell replication, and a high-dispersal regime, where dispersal events largely govern assembly. They proposed that these regimes are separated by the threshold c=r/(2lnK), where the right-hand side corresponds to the inverse of the mean time it takes for the first microbe dispersing from the pool into the community to replicate and reach the carrying capacity before a second dispersal event occurs, assuming logistic within-community growth [23].

In the low-dispersal regime, on average, only a single cluster contributes to community assembly, meaning that only *n* dispersing microbes effectively shape the community. Beyond the low-dispersal regime, assuming equal replication rates for both species (i.e., $r_{A}=r_{B}=r$), we derive the mean number of contributing clusters, denoted by $m_{cluster}$, which reads (S1 Text - Sect 7)

$$m_{cluster}=\frac{c}{r}\log\left(1+\frac{rK}{nc}\right),$$ (1)

while the total number of contributing dispersing microbes is $n\times m_{cluster}$.

To validate these predictions, we simulate community assembly across a range of dispersal rates while keeping the replication rate constant. We quantify both the number of clusters contributing to assembly and the number of dispersing microbes.

**Fig 2:**
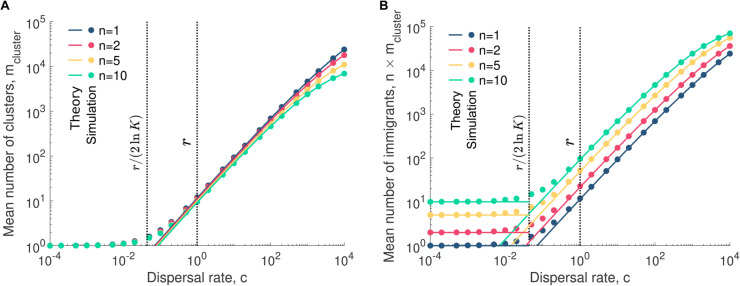
Higher dispersal rates increase the contribution of dispersal to early community assembly. Panels **A** and **B** show the mean number of clusters contributing to community assembly $m_{cluster}$ and the mean number of immigrants (i.e., dispersing microbes) *n* × $m_{cluster}$ as a function of the dispersal rate *c* for various cluster sizes *n*, respectively. In both panels, the simulated data are averaged over 10^4^ microbial communities. The 95% confidence interval bars are smaller than the markers and therefore are not displayed in the figure. The solid lines represent our analytical predictions (see Eq 1). The two vertical dotted lines indicate key dispersal thresholds: c=r/(2lnK), where the mean time between the first and second dispersal events equals the time to reach carrying capacity via replication, and *c* = *r*, where dispersal and replication rates are equal. Parameter values: replication rates $r_{A}=r_{B}=1$, relative abundance of A in the pool $p_{A}=1/2$, carrying capacity *K* = 10^5^.

Fig 2 confirms the accuracy of our predictions. Fig 2A shows that in the low-dispersal regime, only a single cluster contributes to assembly, regardless of its size. Beyond this regime, the number of dispersal events decreases with cluster size, as larger clusters add more microbes per event. Fig 2B indicates that, in the low-dispersal regime, exactly *n* microbes contribute, meaning larger clusters correspond to a larger initial inoculum. Beyond this regime, the number of contributing dispersing microbes increases with cluster size.

These results highlight the relative contributions of dispersal and cell replication to community assembly. By quantifying these contributions, we can determine the extent to which cell replication drives community assembly, which is important to assess how selection will act.

### Cluster dispersal homogenizes microbial communities

To examine the impact of cluster dispersal on microbial community assembly, we first consider the neutral case, where both species share the same replication rate (i.e., $r_{A}=r_{B}=r$). Specifically, we simulate community assembly across various dispersal rates while keeping the replication rate constant and quantify the bimodality coefficient BC of the resulting abundance fluctuation distributions (Fig 1B, 1C, and 1D).

We derive the expression for the bimodality coefficient under the low- and high-dispersal regimes (S1 Text - Sect 1)

$$BC\underset{K\gg 1}{\approx }\left\{ \begin{array}{cc} \frac{(n+2)(n+3)(p_{A}-1)p_{A}\left(1-\frac{9(n+1)(1-2p_{A}{)}^{2}}{2(n+2{)}^{2}(p_{A}-1)p_{A}}\right)}{3(n+1)((n-7)(p_{A}-1)p_{A}-2)} & \text{ if }c\ll r,\\ \frac{1}{3} & \text{ if }c\gg r\;. \end{array}\right.$$ (2)

Assuming that both species are present in equal abundance in the microbial pool (i.e., $p_{A}=1/2$) simplifies the previous equations to

$$BC\underset{K\gg 1}{\approx }\left\{ \begin{array}{cc} \frac{\left(n+2)(n+3\right)}{3(n+1{)}^{2}} & \text{ if }c\ll r,\\ \frac{1}{3} & \text{ if }c\gg r\;. \end{array}\right.$$ (3)

As shown by these equations, the bimodality coefficient does not depend on the carrying capacity (provided K≫1), but only on the cluster size and the species abundances in the microbial pool that determine cluster composition. These equations also show that, in the low-dispersal regime, the bimodality coefficient ranges from 1 when *n* = 1 to 1/3 when n≫1, which is identical to the BC value observed in the high-dispersal regime, where BC = 1/3 for any cluster size. Consequently, large clusters yield the same bimodality coefficient in both assembly regimes, making them indistinguishable through this metric.

**Fig 3:**
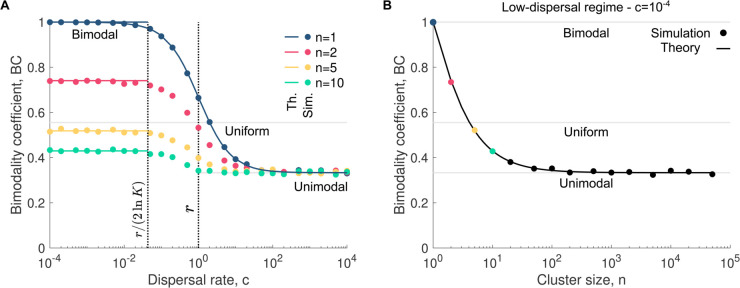
Cluster dispersal blurs the boundary between assembly regimes. Panel **A** shows the bimodality coefficient BC as a function of the dispersal rate *c* for various cluster sizes *n*, whereas panel **B** shows it as a function of the cluster size *n*. In all panels, each data point corresponds to the bimodality coefficient of an abundance fluctuation distribution obtained from 10^4^ simulated microbial communities. The solid lines represent our analytical predictions (see Eq 3). The two vertical dotted lines indicate key dispersal thresholds: c=r/(2lnK), where the mean time between the first and second dispersal events equals the time to reach carrying capacity via replication, and *c* = *r*, where dispersal and replication rates are equal. Parameter values: replication rates $r_{A}=r_{B}=1$, dispersal rate *c* = 10^−4^ (in **B**), relative abundance of A in the pool $p_{A}=1/2$, carrying capacity *K* = 10^5^.

Fig 3A shows the bimodality coefficient as a function of the dispersal rate. As expected, BC approaches 1/3 at high dispersal rates, indicating a unimodal abundance fluctuation distribution, characteristic of high richness and low between-community dissimilarity. This behavior is independent of cluster size, as dispersal in this regime homogenizes community composition to reflect that of the microbial pool.

In contrast, in the low-dispersal regime, the bimodality coefficient depends strongly on cluster size, as shown in Fig 3B. When cluster size is small (e.g., *n* = 1), communities are typically populated by a single species, leading to a BC of 1, signifying low richness and high between-community dissimilarity. In other words, high values of BC arise because small cluster are often *monochromatic*, containing only one species. As cluster size increases, the probability of introducing multiple species in the initial dispersal event rises. This increases richness, decreases between-community dissimilarity and, thus, reduces the bimodality coefficient toward 1/3. For example, if $p_{A}=1/2$, the probability that a cluster of size 2 contains only one species is 1/2, while for size 10, it drops to 1/512. Therefore, larger clusters promote more diverse initial dispersal events, shifting community assembly dynamics toward those observed in the high-dispersal regime (see also S2 Fig.).

It is important to note that accounting for the dispersal of clusters is not equivalent to increasing the dispersal rate of individual microbes. Even when the bimodality coefficient is plotted as a function of the effective dispersal rate, that is, the number of individual microbes dispersing per unit time rather than the number of clusters, its maximum value still decreases with increasing cluster size (S3 Fig.). This is because larger clusters are more likely to introduce multiple species simultaneously.

Eq 2 and Fig 3B together provide a useful framework for determining whether a given cluster size homogenizes microbial communities and supports distinguishable assembly regimes.

### Cluster dispersal modulates the impact of local selection on microbial community assembly

A second metric for characterizing microbial community assembly is the mean relative abundance. The mean relative abundance quantifies the extent to which a community is dominated by a single species. Thus, this metric can be used to determine, for example, if local selection is present, i.e., whether two species have different replication rates [18]. Whereas in the previous section we focused on the neutral case (i.e., $r_{A}=r_{B}$), we now assume that the two species have different replication rates (i.e., $r_{A}\neq r_{B}$), resulting in a nonzero selection coefficient $s=r_{A}-r_{B}$, and investigate the impact of within-community selection on the mean relative abundance. Note that incorporating within-community selection has little impact on the bimodality coefficient, which remains similar to that obtained in the neutral case (S4 Fig.).

Analytically, we build on [26]’s work and derive an equation giving the mean absolute abundance of A once the community assembly is complete, denoted by $\langle N_{A}\rangle$. Note that here the mean relative abundance is simply given by the mean absolute abundance of A divided by the carrying capacity once the community assembly is complete. In the low-dispersal regime, we obtain the following expression (S1 Text - Sect 1)

$$\langle N_{A}\rangle \underset{s\ll 1}{\approx }\frac{p_{A}K\left(1+n+(-1+n+p_{A}-np_{A})s\log\left(\frac{K}{n}\right)\right)}{n+1},$$ (4)

which, assuming that both species are present in equal abundance in the pool (i.e., $p_{A}=1/2$), reduces to

$$\langle N_{A}\rangle \underset{s\ll 1}{\approx }\frac{1}{4}K\left(\frac{(n-1)s\log\left(\frac{K}{n}\right)}{n+1}+2\right)\;.$$ (5)

If the selection coefficient is zero, the mean absolute abundance is equal to $p_{A}K$ for any cluster size. This value is also obtained for a cluster size of 1 or *K* for any selection coefficient. If the selection coefficient is nonzero, the mean absolute abundance varies non-monotonically with increasing cluster size, reaching a maximum that exceeds 50% when *s* > 0 and a minimum below 50% when *s* < 0. In the high-dispersal regime, the community structure is expected to reflect that of the microbial pool such that the mean absolute abundance satisfies $\langle N_{A}\rangle =p_{A}K$.

**Fig 4:**
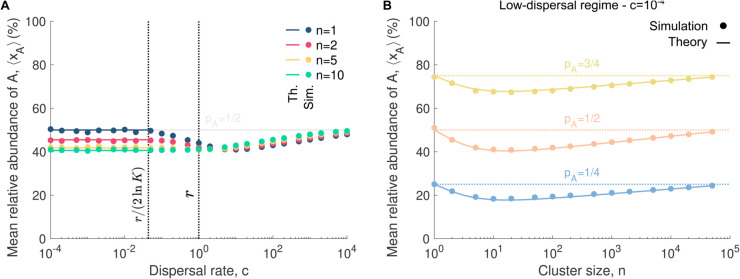
Cluster dispersal and within-community selection alter species abundance in a non-monotonic way. Panel **A** shows the mean relative abundance of A $\langle x_{A}\rangle =\langle N_{A}\rangle /K$ as a function of the dispersal rate *c* for various cluster sizes *n*, whereas panel **B** shows it as a function of the cluster size *n* for different abundances in the pool $p_{A}$ in the low-dispersal regime. In both panels, the simulated data are averaged over 10^4^ microbial communities, whereas the solid lines represent our analytical predictions (Eqs 4 and 5). The 95% confidence interval bars are smaller than the markers and therefore are not displayed in the figure. In Panel **A**, the two vertical dotted lines indicate key dispersal thresholds: c=r/(2lnK), where the mean time between the first and second dispersal events equals the time to reach carrying capacity via replication, and *c* = *r*, where dispersal and replication rates are equal. In both panels, the horizontal lines show the relative abundance of species A in the microbial pool. Parameter values: replication rate of A $r_{A}=1$, replication rate of B $r_{B}=1.05$, dispersal rate *c* = 10^−4^ (in **B**), relative abundance of A in the pool $p_{A}=1/2$ (in **A**), carrying capacity *K* = 10^5^.

We validate our predictions by simulating microbial community assembly, assuming $r_{A}=1$ and $r_{B}=1.05$, which corresponds to a selection coefficient of *s* = −0.05. Fig 4A shows the mean relative abundance of species A as a function of dispersal rate for various cluster sizes. In the high-dispersal regime, where community assembly is governed solely by dispersal, the mean relative abundance converges to that of the microbial pool, rendering replication rates insignificant. Similarly, in the low-dispersal regime, this convergence occurs when the cluster size is 1, as the first dispersing individual populates the entire community before a subsequent dispersal event, making replication rates inconsequential [18]. However, for cluster sizes greater than 1, dispersal introduces multiple species, leading to competitive dynamics when replication rates differ. As a result, the mean relative abundance, given by Eq 4, deviates from that of the microbial pool.

Fig 4B depicts the mean relative abundance of species A as a function of cluster size in the low-dispersal regime, revealing the non-monotonic pattern described above, with an extremum at intermediate cluster sizes. For clusters of size 1, the probability of containing an A microbe is simply $p_{A}$, and this single microbe populates the community without competition. Conversely, large clusters, which contain on average $p_{A}$ × *n* A microbes dominate microbial community assembly due to their size, leaving limited opportunities for cell replication. In both extreme cases, replication rate differences become negligible. However, at intermediate cluster sizes, dispersal introduces a mix of species, leading to competition that amplifies the effects of differential replication rates, driving the observed non-monotony. These dynamics underscore the critical role of cluster dispersal in shaping community structure within the low-dispersal regime.

In summary, when cluster size equals one, selection influences community assembly only at intermediate dispersal rates. However, as soon as clusters contain more than one individual, selection also becomes relevant in the low-dispersal regime—unless the clusters are excessively large. Thus, cluster dispersal modulates the extent to which selection shapes microbial community assembly by influencing species abundance.

### Bimodality coefficient and species abundance shed light on assembly regime, within-community selection and cluster dispersal

Previous work has demonstrated that the bimodality coefficient and mean relative abundance can serve as metrics to characterize microbial community assembly under varying dispersal rates [15,18]. Specifically, the bimodality coefficient helps determine whether assembly is driven by cell replication, dispersal events, or both, while mean relative abundance reflects differences in microbial traits, such as replication rates.

**Fig 5:**
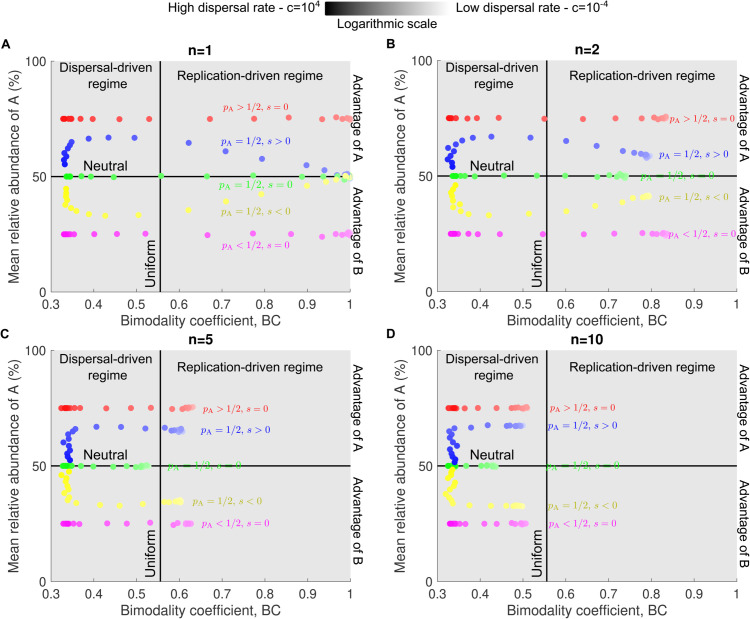
Bimodality coefficient and mean relative abundance across multiple dispersal rates reveal patterns of selection and cluster dispersal. In all panels, each data point shows the mean relative abundance of species A and the bimodality coefficient obtained from an abundance fluctuation distribution simulated over 10^4^ microbial communities. Illustrative examples of these distributions for different parameter regimes are provided in S2 Fig. Each panel corresponds to a different cluster size *n*. Colors correspond to dispersal rate, with darker shades indicating higher dispersal. The bar denotes the gradient of dispersal rates from low (light) to high (dark). Parameter values: replication rate of A $r_{A}=1$ (red, green, yellow, purple) $r_{A}=1.1$ (blue), replication rate of B $r_{B}=1$ (red, blue, green, purple) $r_{B}=1.1$ (yellow), relative abundance of A in the pool $p_{A}=1/2$ (blue, green, yellow) $p_{A}=3/4$ (red) $p_{A}=1/4$ (purple), carrying capacity *K* = 10^5^, dispersal rate $c=10^{-4}-10^{4}$.

Figs 5 and S5 show the mean relative abundance of type A plotted against the bimodality coefficient in a series of panels, each representing a different cluster size *n*. In each panel, five datasets are displayed, corresponding to different combinations of microbial pool abundances $p_{A}$ and selection coefficients $s=r_{A}$ − $r_{B}$. Within each dataset, individual points indicate results from simulations with different dispersal rates *c*. Regardless of the microbial pool or selection coefficient, the bimodality coefficient decreases to 1/3 as the dispersal rate increases. The mean relative abundance provides insights into the values of $p_{A}$ and *s*. When selection is present, that is, when the selection coefficient satisfies s≠0, the mean relative abundance of species A displays an extremum. Specifically, when species A is beneficial (i.e., *s* > 0), the curve exhibits a maximum, while when it is deleterious (i.e., *s* < 0), it shows a minimum. In contrast, under neutral conditions (i.e., *s* = 0), the relationship is flat. This effect is most pronounced when clusters are very small relative to the carrying capacity. In this case, mean relative abundance first increases (respectively decreases) with dispersal before reversing and forming an extremum. For large clusters, by contrast, the mean relative abundance is already close to its maximum (or minimum) at low dispersal rates, and then shifts monotonically as dispersal increases. Moreover, if species A is more prevalent in the microbial pool, its mean relative abundance exceeds 50% in the high-dispersal regime. If it is less prevalent, the mean falls below 50% (see S8 Fig. for the low-dispersal regime).

**Fig 6:**
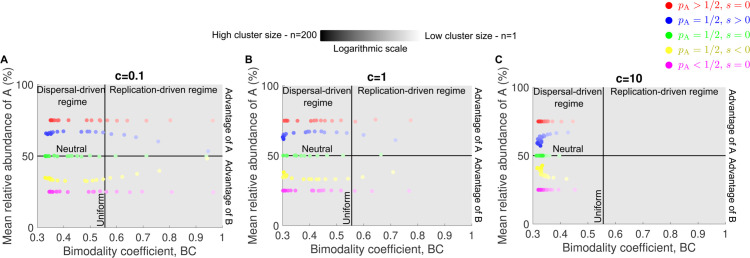
Bimodality coefficient and mean relative abundance across multiple cluster sizes reveal patterns of selection. In all panels, each data point shows the mean relative abundance of species A and the bimodality coefficient obtained from an abundance fluctuation distribution simulated over 10^4^ microbial communities. Illustrative examples of these distributions for different parameter regimes are provided in S2 Fig. Each panel corresponds to a different dispersal rate *c*. Colors correspond to cluster size, with darker shades indicating higher cluster size. The bar denotes the gradient of cluster sizes from low (light) to high (dark). Parameter values: replication rate of A $r_{A}=1$ (red, green, yellow, purple) $r_{A}=1.1$ (blue), replication rate of B $r_{B}=1$ (red, blue, green, purple) $r_{B}=1.1$ (yellow), relative abundance of A in the pool $p_{A}=1/2$ (blue, green, yellow) $p_{A}=3/4$ (red) $p_{A}=1/4$ (purple), carrying capacity *K* = 10^5^, cluster size *n* = 1−200.

Figs 6 and S6 present the same data as in Figs 5 and S5, but organized by dispersal rate *c* rather than cluster size *n*. This alternate view reveals the same patterns of selection and microbial pool effects described above, with the mean relative abundance of type A varying predictably with the bimodality coefficient depending on $p_{A}$ and *s*.

Importantly, we show that both metrics, the bimodality coefficient and mean relative abundance, are influenced by the number of microbes dispersing simultaneously, i.e., cluster size. In particular, as cluster size increases, the maximum value of the bimodality coefficient decreases (Fig 3), whereas its effect on mean relative abundance is non-monotonic (Fig 4). Nonetheless, our results demonstrate that, across different dispersal rates and cluster sizes, these two metrics still reveal patterns that distinguish whether two species differ in abundance within the microbial pool and exhibit varying replication rates (Figs 5, S5, 6, and S6). When cluster size is large, a higher number of data points is required to avoid misidentifying, for example, cases ($p_{A}=1/2$, *s* < 0) and ($p_{A}<1/2$, *s* = 0). Fig 5C and 5D illustrate a case where mean relative abundance remains relatively constant across a wide range of dispersal rates, even when the selection coefficient is nonzero, a pattern not observed for small cluster sizes (Fig 5A and 5B).

### Our predictions remain valid with multiple species

So far, we have considered that the microbial pool contains only two species, which is relevant to some experimental data [15,21,22]. However, in the wild, microbial communities are likely to be made up of multiple species. This leads us to extend our model to *S* species, assuming they all have the same replication rate *r*. These species are denoted by i=1,2,...,S and are each present in the microbial pool in abundance 1/*S*. Since the bimodality coefficient is not suitable for cases with more than two species, we use *α*- and *β*-diversity to describe the abundance fluctuation distributions.

Here, *α*-diversity is measured as species richness, i.e., the total number of microbial species present in a community when its size reaches the carrying capacity. We note that other metrics, such as the Shannon or Simpson indices, are commonly used to quantify *α*-diversity, but for simplicity we focus on species counts in this study. In the high-dispersal regime, richness is expected to equal the number of species in the pool, provided that the total number of species does not exceed the community’s carrying capacity. In the low-dispersal regime, richness is equal to that of the first cluster dispersing from the pool, assuming no mortality. Otherwise, richness would correspond to that of the first cluster that successfully establishes in the community. The probability that a cluster of size *n* has a richness of $\alpha_{R}$ is given by (S1 Text - Sect 6)

P(αR)=1Sn−1(∑k=1αR(αRk)(−1)αR−kkn)(∏q=1αR−1S−j)αR!, (6)

which allows us to derive the mean value of richness $\langle \alpha_{R}\rangle =\sum_{\alpha_{R}=1}^{S}\alpha_{R}P(\alpha_{R})$.

**Fig 7:**
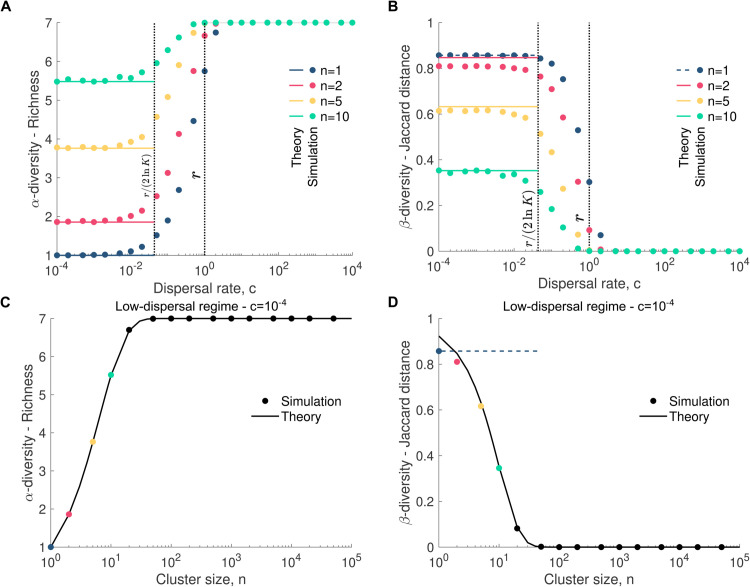
Cluster dispersal increases *α*-diversity and decreases *β*-diversity. Panels **A** and **C** show the richness as a function of the dispersal rate and cluster size, respectively, where **C** focuses on the low-dispersal regime. The simulated data are averaged over 10^3^ stochastic replicates (i.e., microbial communities). The 95% confidence interval bars are smaller than the markers and therefore are not displayed in the figure. Panels **B** and **D** represent the Jaccard distance as a function of the dispersal rate and cluster size, respectively, where **D** focuses on the low-dispersal regime. The data points are averaged over the comparison of each pair of 10^3^ stochastic replicates (i.e., microbial communities). The solid lines represent our analytical predictions (Eqs 6 and 9). In panels **A** and **B**, the two vertical dotted lines correspond to key dispersal thresholds: c=r/(2lnK), which approximates the mean time between the first dispersal event and reaching carrying capacity through replication alone [18], and *c* = *r*, where the dispersal rate equals the replication rate. In panels **B** and **D**, the dashed line represents the exact Jaccard distance for *n* = 1 (i.e., $\langle \beta_{J}\rangle =1$ − 1/S). Parameter values: replication rate *r* = 1, dispersal rate *c* = 10^−4^ (in **C** and **D**), number of species *S* = 7, relative abundance of each species in the pool *p* = 1/*S*, carrying capacity *K* = 10^5^.

To validate our analytical predictions, we generated *in silico* data by simulating the assembly of microbial communities from a pool containing seven neutral species. We then calculated their richness (Fig 7A and 7C). As shown in Fig 7A, richness increases with dispersal rate, regardless of cluster size. However, while high dispersal rates yield the same richness across cluster sizes, at low dispersal rates, larger clusters exhibit higher richness. In the low-dispersal regime, the richness of a microbial community reflects that of the first microbial cluster, whose composition may differ from the pool. Naturally, larger clusters may contain more species, leading to increased richness. In contrast, in the high-dispersal regime, microbial community composition mirrors that of the pool, resulting in uniform richness across cluster sizes.

In addition to *α*-diversity, we also quantify *β*-diversity, which determines the dissimilarity of two microbial communities. Here, we focus on the Jaccard distance, which quantifies the proportion of species that differ between two communities. Mathematically, the Jaccard distance is defined as $\beta_{J}=1-J(X,Y)$, where J(X,Y) is the Jaccard similarity coefficient. This coefficient is equal to |X∩Y|/(|X|+|Y|−|X∩Y|), where |X| and |Y| are the richness of communities X and Y, respectively, and |X∩Y| the number of shared species between communities X and Y. A Jaccard distance of 0 indicates identical composition, while a value of 1 signifies completely distinct communities.

In the high-dispersal regime, dispersal tends to homogenize community composition, resulting in a Jaccard distance close to zero. In contrast, in the low-dispersal regime, the Jaccard distance is influenced by the composition of the initial clusters that populate each community. Assuming that *p*_*i*_ = 1/*S* for 1≤i≤S, we can show the probability that two communities have a number of shared species equal to |X∩Y|=k is approximately given by (S1 Text - Sect 6)

P(k)≈(Sk)qk(1−q)S−k, (7)

where

$$q={\left(1-{\left(1-\frac{1}{S}\right)}^{n}\right)}^{2},$$ (8)

is the probability that a given species is present in both clusters and, thus, in both communities. This approximation treats the presence of each species in both clusters as independent Bernoulli trials with probability *q*. Eq 7 allows us to compute the mean number of shared species $\langle k\rangle =\sum_{k=1}^{S}kP(k)$. Thus, the mean Jaccard distance is approximately given by

$$\langle \beta_{J}\rangle \approx 1-\frac{\langle k\rangle }{2\langle \alpha_{R}\rangle -\langle k\rangle },$$ (9)

where we assumed that |X| and |Y| are simply equal to the mean richness $\langle \alpha_{R}\rangle$ (Fig 7A and 7C).

In the case *n* = 1, where microbes disperse individually, the Jaccard distance can be computed exactly. In the low-dispersal regime, each community contains a single species, so the number of shared species between two communities is either 0 or 1. The probability that both communities contain the same species is 1/*S*, and therefore the mean Jaccard distance is $\langle \beta_{J}\rangle =1-1/S$.

Using the microbial communities generated previously, we compare each pair of them and compute their *β*-diversity. Fig 7 shows that *β*-diversity decreases with dispersal rate. In the high-dispersal regime, dispersal homogenizes microbial communities so they all have compositions similar to the microbial pool. In the low-dispersal regime, the larger cluster size, the lower the *β*-diversity, as they likely introduce multiple species into microbial communities (see Fig 7D).

## Discussion

In this work, we built a microbial community assembly model to examine the impact of cluster dispersal on community structure during the early stages of assembly. Our model accounts for two events: dispersal and within-community replication (Fig 1). The timescales associated with these two events strongly impact richness and dissimilarity during the stochastic assembly of microbial communities. Specifically, [15] and [18]’s work shed light on distinct assembly regimes: one driven by high dispersal rates relative to replication rates that homogenizes microbial communities, thus reducing between-community diversity while increasing richness. Conversely, low dispersal rates relative to replication rates are a barrier to high richness and lead to dissimilar structures. Here, we showed that cluster dispersal mitigates the latter effect. Specifically, clusters, when large, likely introduce multiple species into local communities at a time. Thus, even in the low-dispersal regime, dispersal can contribute to homogenizing microbial communities (Fig 3), showing that timescales are not the only parameter to consider when assessing the impact of dispersal on richness and between-community dissimilarity.

It is widely accepted that very low dispersal rates can lead to dispersal limitation, resulting in highly dissimilar microbial community structures [14,27,28]. Conversely, very high dispersal rates promote homogenization, leading to more similar community structures [14,27,28]. However, our study revealed that even at very low dispersal rates, microbial communities can become less dissimilar if microbes disperse in large clusters. This suggests that dispersal plays an increasingly significant role as cluster size grows, regardless of dispersal rate, underscoring cluster size as a key factor in microbial community assembly and diversity.

[18] demonstrated how the bimodality coefficient and mean relative abundance can be used as metrics to identify assembly regimes and compare microbial traits across species in experimental data under various dispersal rates and cluster sizes. Here, we extended their approach to scenarios beyond the dispersal of individual microbes (Figs 5 and 6). Specifically, while our results confirm that these two metrics consistently distinguish whether two species differ in abundance within the microbial pool or exhibit varying replication rates, a larger number of data points is required to prevent misidentification when cluster sizes are large.

In S1 Text (Sect 10) and S9 Fig., we applied our model predictions to a reanalysis of the experimental data set obtained by [15] to illustrate their practical utility. To our knowledge, this is the only data set in which multiple dispersal rates or cluster sizes were tested, a key requirement for detecting signals of selection between strains or species. Other studies on community assembly from initially germ-free hosts exist, such as [21], but they did not explore multiple dispersal rates or cluster sizes. Specifically, we demonstrated how the bimodality coefficient and mean relative abundance can help identify different assembly regimes and microbial trait differences. While our predictions are not perfect, they revealed some signals of selection between strains and species. It is important to note that, in our model, relative and absolute abundances are equivalent because the final community size always reaches the carrying capacity. In experimental settings, obtaining absolute abundance estimates can be challenging, and our model does not account for experimental noise inherent to dilution and plating methods, which has been shown to influence observed community composition [29]. Importantly, our study, together with those of [15] and [18], highlights that diversity patterns strongly depend on how dispersal is experimentally implemented, emphasizing the need for careful experimental design when investigating community assembly.

Although multiple community assembly experiments involve two species [15,21,22], natural microbial communities likely harbor many more species. This led us to extend our model to *S* species. Since the bimodality coefficient is not suitable for cases with more than two species, we derived analytical predictions for *α*- and *β*-diversity, which allowed us to show that our results drawn for two species still hold for *S* species (Fig 7). Specifically, cluster dispersal homogenizes microbial communities increasing their richness and decreasing their between-community dissimilarity.

Our results resonate with recent [30]’s experimental findings on dose-dependent colonization in microbial coalescence experiments. In our study and theirs, the initial size of dispersal events, whether clusters in our model or propagule size *in vitro*, strongly influences community assembly and diversity patterns. A key difference between the two studies is that [30] explicitly investigated the impact of resource competition, whereas our current model does not include such competition.

Although our model relies on simplifying assumptions, we confirmed in S1 Text (Sect 8) and S8 Fig. that our main conclusions are robust when these assumptions are relaxed. Specifically, we showed that introducing dispersal without saturation (i.e., applying the logistic term (1 − N/K) only to the replication rate), incorporating cell death, or allowing random cluster sizes does not qualitatively alter the diversity patterns we reported.

In summary, our work underscores the crucial role of cluster dispersal in the assembly of microbial communities, not only by influencing richness and between-community dissimilarity, but also by modulating the extent to which selection shapes community structure.

## Supporting information

S1 Text Formal analysis. 1.1 Low-dispersal regime. 1.2 High-dispersal regime. 1.3 Both regimes in the neutral case for a cluster size of 1. 2 Extension to *S* species. 2.1 *α*-diversity. 2.1.1 Richness. 2.1.2 High-dispersal regime. 2.1.3 Low-dispersal regime. 2.2 *β*-diversity. 2.2.1 Jaccard distance. 2.2.2 High-dispersal regime. 2.2.3 Low-dispersal regime. 3 Mean number of clusters contributing to community assembly. 4 Robustness analyses. 4.1 Dispersal without saturation. 4.2 Community assembly with cell death. 4.3 Random cluster size. 5 Experimental data analysis.
(PDF)

S2 FigCluster dispersal homogenizes microbial communities.Each panel shows the number of communities as a function of the relative abundance of species A for a given pair (*n*,*c*), where *n* is the cluster size and *c* is the dispersal rate. Parameter values: $r_{A}=r_{B}=1$, *K* = 10^5^, $p_{A}=1/2$, number of communities  = 10^3^.(EPS)

S3 FigConsidering cluster dispersal is not equivalent to increasing the dispersal rate of individual microbes.Bimodality coefficient (BC) as a function of the effective dispersal rate *n* × *c* for various cluster sizes *n*. Each data point corresponds to 10^4^ simulated communities. Solid lines show analytical predictions (Eq 3). Vertical dotted lines indicate c=r/(2lnK) and *c* = *r*. Parameter values: $r_{A}=r_{B}=1$, $p_{A}=1/2$, *K* = 10^5^.(EPS)

S4 FigSelection induces bimodality coefficient values similar to those observed under neutrality.BC as a function of dispersal rate *c* for various cluster sizes *n*. Each data point corresponds to 10^4^ simulated communities. Solid lines show analytical predictions (Eq 3). Parameter values: $r_{A}=1$, $r_{B}=1.05$, $p_{A}=1/2$, *K* = 10^5^.(EPS)

S5 FigBimodality coefficient and mean relative abundance across multiple dispersal rates reveal patterns of selection and cluster dispersal.Each data point shows the mean relative abundance of species A and BC from 10^4^ simulations. Panels correspond to different cluster sizes *n*. Colors indicate dispersal rate. Parameter values include $r_{A}=1$ or 1.1, $r_{B}=1$ or 1.1, $p_{A}=1/4$ or 3/4, *K* = 10^5^, *c* = 10^−4^–10^4^.(EPS)

S6 FigBimodality coefficient and mean relative abundance across multiple cluster sizes reveal patterns of selection.Each panel corresponds to a different dispersal rate *c*. Colors indicate cluster size *n*. Each data point corresponds to 10^4^ simulated communities. Parameter values: $r_{A}=1$ or 1.1, $r_{B}=1$ or 1.1, $p_{A}=1/4$ or 3/4, *K* = 10^5^, *n* = 1–200.(EPS)

S7 FigResults remain robust under relaxed model assumptions.Panels A, C, E show BC as a function of *c*; panels B, D, F as a function of *n*. Each data point corresponds to 10^4^ simulated communities. Solid lines show analytical predictions (Eq 3). Parameter values include $r_{A}=r_{B}=1$, *d* = 0.1 where indicated, $p_{A}=1/2$, *K* = 10^5^.(EPS)

S8 FigThe relative abundance of species can differ from the relative pool abundance under selection in the low-dispersal regime.Heatmaps show $\langle N_{A}\rangle /K$ as a function of selection coefficient *s* and pool abundance $p_{A}$. Computed from Eq 4. Parameter values: *K* = 10^5^.(EPS)

S9 Fig Bimodality coefficient and mean relative abundance help to analyze experimental data. Mean relative abundance of *E. coli* (dsRed) and *E. aerogenes* as a function of BC for different worm strains. Error bars represent 95% confidence intervals. Experimental data from [15].
(EPS)
