# Supplementary material for: Cluster dispersal shapes microbial diversity during community assembly
Source: PLoS Comput Biol. 2026 Feb 2;22(2):e1013918. doi: 10.1371/journal.pcbi.1013918 (PMC12885383; doi:10.1371/journal.pcbi.1013918)
Supplement: S1 Text — 1.1 Low-dispersal regime. 1.2 High-dispersal regime. 1.3 Both regimes in the neutral case for a cluster size of 1. 2 Extension to S species. 2.1 α-diversity. 2.1.1 Richness. 2.1.2 High-dispersal regime. 2.1.3 Low-dispersal regime. 2.2 β-diversity. 2.2.1 Jaccard distance. 2.2.2 High-dispersal regime. 2.2.3 Low-dispersal regime. 3 Mean number of clusters contributing to community assembly. 4 Robustness analyses. 4.1 Dispersal without saturation. 4.2 Community assembly with cell death. 4.3 Random cluster size. 5 Experimental data analysis. (PDF) [file pcbi.1013918.s001.pdf]

Supporting Information

Loïc Marrec*[1,2] and Sonja Lehtinen[1,2]

[1] *Département de Biologie Computationnelle, Université de Lausanne, Lausanne, Switzerland*
[2] *Swiss Institute of Bioinformatics, Lausanne, Switzerland*

Contents

1 Formal analysis **2**
1.1 Low-dispersal regime 2
1.2 High-dispersal regime 3
1.3 Both regimes in the neutral case for a cluster size of 1 4

2 Extension to S species **5**
2.1 α-diversity 5
2.1.1 Richness 5
2.1.2 High-dispersal regime 5
2.1.3 Low-dispersal regime 5
2.2 β-diversity 6
2.2.1 Jaccard distance 6
2.2.2 High-dispersal regime 6
2.2.3 Low-dispersal regime 7

3 Mean number of clusters contributing to community assembly **8**

4 Robustness analyses **9**
4.1 Dispersal without saturation 9
4.2 Community assembly with cell death 9
4.3 Random cluster size 9

5 Experimental data analysis **10**

*Corresponding author: loic.marrec@unil.ch

1 Formal analysis

The assembly of a microbial community is a stochastic process that involves, in our model, two events: dispersal of a cluster from the microbial pool to the community, and intra-community cell replication. As the community is initially microbe-free, its assembly starts with small sizes. A deterministic formalism, based on a system of ordinary differential equations, does not adequately capture the stochastic dynamics of community growth involving low sizes [1].

To address this, we adopt a microscopic, probabilistic description of microbial community assembly. More specifically, we formulate a system of equations that governs the dynamics of the probability of having N_A microbes of strain A in a community of size N (and thus $N_\mathrm{B} = N - N_\mathrm{A}$ microbes of strain B), denoted by $P(N_\mathrm{A}, N)$.

We distinguish between two regimes: the low- and high-dispersal regimes.

1.1 Low-dispersal regime

In the low-dispersal regime, a first cluster of size n disperses and assembly is then completed by cell replication. This regime leads to the following system of equations [2]

$$P(N_\mathrm{A}, N+1) = \alpha_N^{N_\mathrm{A}-1} P(N_\mathrm{A}-1, N) + (1-\alpha_N^{N_\mathrm{A}}) P(N_\mathrm{A}, N)\,, \tag{S1}$$

with $N_{\mathrm{A},0}$ as an initial condition, which is drawn from the binomial dsitribution $\mathcal{B}(n, p_\mathrm{A})$. The function $\alpha_N^{N_\mathrm{A}}$ denotes the probability that the increase in community size by one microbe ($N \to N+1$) is due to the replication of an A microbe. This probability is given by [2]

$$\alpha_N^{N_\mathrm{A}} = \frac{r_\mathrm{A} N_\mathrm{A}}{r_\mathrm{A} N_\mathrm{A} + r_\mathrm{B}(N - N_\mathrm{A})}\,. \tag{S2}$$

We are interested in deriving the moments of N_A, i.e., $\langle N_\mathrm{A}^k \rangle = \sum_{N_\mathrm{A}=0}^{N} N_\mathrm{A}^k P(N_\mathrm{A}, N)$. They can be calculated by multiplying Equation S1 by N_A^k, summing over the possible values of N_A, and solving the resulting recurrence relation . Assuming the neutral case ($r_\mathrm{A} = r_\mathrm{B} = r$) simplifies the derivation of the moments, which leads to

$$\langle N_\mathrm{A} \rangle = p_\mathrm{A} N\,, \tag{S3}$$

$$\langle N_\mathrm{A}^2 \rangle \underset{N\gg1}{\approx} \frac{(2+(n-1)p_\mathrm{A})p_\mathrm{A}N^2}{n+1}\,, \tag{S4}$$

$$\langle N_\mathrm{A}^3 \rangle \underset{N\gg1}{\approx} \frac{(6+(n-1)p_\mathrm{A}(6+(n-2)p_\mathrm{A}))p_\mathrm{A}N^3}{(n+1)(n+2)}\,, \tag{S5}$$

and

$$\langle N_\mathrm{A}^4 \rangle \underset{N\gg1}{\approx} \frac{(24+(n-1)p_\mathrm{A}(36+(n-2)p_\mathrm{A}(12+(n-3)p_\mathrm{A})))p_\mathrm{A}N^4}{(n+1)(n+2)(n+3)}\,. \tag{S6}$$

Note that these moments were derived by first calculating the moments of $N_{\mathrm{A},0}$ (e.g., $\langle N_{\mathrm{A},0} \rangle = p_\mathrm{A} n$). Now we can derive the variance, skewness, and kurtosis, which are given by

$$\sigma^2 \underset{N\gg1}{\approx} \frac{2(1-p_\mathrm{A})p_\mathrm{A}N^2}{n+1}\,, \tag{S7}$$

$$\gamma \underset{N\gg1}{\approx} \frac{3(1-2p_\mathrm{A})}{(n+2)\sqrt{\frac{2(1-p_\mathrm{A})p_\mathrm{A}}{1+n}}}\,, \tag{S8}$$

and

$$\kappa \underset{N\gg1}{\approx} \frac{(n+1)\left(p_\mathrm{A}\left(n^2 p_\mathrm{A}(p_\mathrm{A}(3p_\mathrm{A}-1)-1) + 2n(p_\mathrm{A}-1)(5p_\mathrm{A}-7) - 6(13(p_\mathrm{A}-2)p_\mathrm{A}+17)\right)+24\right)}{4(n+2)(n+3)(1-p_\mathrm{A})^2 p_\mathrm{A}}\,. \tag{S9}$$

The skewness and kurtosis allow us to obtain the bimodality coefficient [3, 4]

$$\mathrm{BC} = \frac{\gamma^2 + 1}{\kappa} \,. \tag{S10}$$

Although we have an explicit formula for the bimodality coefficient, it is too complex to be written here.

If both species have distinct replication rates ($r_\mathrm{A} \neq r_\mathrm{B}$), resulting in a nonzero selection coefficient ($s \neq 0$), the moments are more difficult to obtain. To get around this difficulty, we approximate Equation S1 by assuming that N_A and N are continuous variables. This assumption allows us to reduce Equation S1 to the partial differential equation [2]

$$\frac{\partial P(N_\mathrm{A}, N)}{\partial N} + \frac{\partial}{\partial N_\mathrm{A}} \left(\alpha_N^{N_\mathrm{A}} P(N_\mathrm{A}, N) \right) = 0 \,. \tag{S11}$$

The moments $\langle N_\mathrm{A}^k \rangle$ can be derived from the previous equation. The neutral case ($r_\mathrm{A} = r_\mathrm{B}$) leads to [2]

$$\langle N_\mathrm{A}^k \rangle_\emptyset = \int_0^N N_\mathrm{A}^k P(N_\mathrm{A}, N) \mathrm{d}N_\mathrm{A} = \frac{(N_{\mathrm{A},0})_k}{(n)_k} N^k \,. \tag{S12}$$

where $\emptyset$ indicates the neutral case, $N_{\mathrm{A},0}$ denotes the number of A microbes in the first cluster, and $(x)_p = x(x+1)...(x+p-1)$ designates the Pochhammer symbol. To obtain the moments for nonzero selection coefficients, we apply a perturbation method, which, to the first order in s ($s \ll 1$), yields

$$\langle N_\mathrm{A}^k \rangle = \sum_{N_{\mathrm{A},0}=0}^{n} \binom{n}{N_{\mathrm{A},0}} p_\mathrm{A}^{N_{\mathrm{A},0}} (1-p_\mathrm{A})^{n-N_{\mathrm{A},0}} \langle N_\mathrm{A}^k \rangle_\emptyset \left[1 + k \frac{n - N_{\mathrm{A},0}}{n+k} \log\left(\frac{N}{n} \right) s \right] . \tag{S13}$$

As done previously, we can obtain the variance, skewness, and kurtosis from the moments, and then the bimodality coefficient.

1.2 High-dispersal regime

In the high-dispersal regime, microbial community assembly is achieved only by dispersal events, without any cell replication. In this regime, a community reaches a size N through the dispersal of q clusters of size n, where $q = N/n$. Thus, the number of A microbes in the final structure of a community is equal to the sum of q random variables that are independently drawn from the binomial distribution $\mathcal{B}(n, p_\mathrm{A})$. The sum of q random variables that are drawn from the binomial distribution $\mathcal{B}(n, p_\mathrm{A})$ is itself drawn from the binomial distribution $\mathcal{B}(q \times n, p_\mathrm{A})$. Thus, the probability of having N_A A microbes in a community of size N satisfies

$$P(N_\mathrm{A}, N) = \binom{N}{N_\mathrm{A}} p_\mathrm{A}^{N_\mathrm{A}} (1-p_\mathrm{A})^{N-N_\mathrm{A}} \,, \tag{S14}$$

where N must be a multiple of n, otherwise the probability is zero. The first four moments satisfy

$$\langle N_\mathrm{A} \rangle = p_\mathrm{A} N \,, \tag{S15}$$

$$\langle N_\mathrm{A}^2 \rangle = p_\mathrm{A}(N + (N-1)N p_\mathrm{A}) \,, \tag{S16}$$

$$\langle N_\mathrm{A}^3 \rangle = N p_\mathrm{A}((N-1)p_\mathrm{A}((N-2)p_\mathrm{A} + 3) + 1) \,, \tag{S17}$$

and

$$\langle N_\mathrm{A}^4 \rangle = N p_\mathrm{A}((N-1)p_\mathrm{A}((N-2)p_\mathrm{A}((N-3)p_\mathrm{A} + 6) + 7) + 1) \,. \tag{S18}$$

From the first four moments, we can derive the variance, skewness, and kurtosis, which are respectively given by

$$\sigma^2 = (1 - p_\mathrm{A}) p_\mathrm{A} N\,, \tag{S19}$$

$$\gamma = \frac{1 - 2p_\mathrm{A}}{\sqrt{(1 - p_\mathrm{A}) p_\mathrm{A} N}}\,, \tag{S20}$$

and

$$\kappa = 3 - \frac{6}{N} + \frac{1}{(1 - p_\mathrm{A}) p_\mathrm{A} N}\,. \tag{S21}$$

The previous quantities allow us to derive an equation for the bimodality coefficient

$$\mathrm{BC} = \frac{1 + (N-4)(1 - p_\mathrm{A}) p_\mathrm{A}}{1 + 3(N-2)(1 - p_\mathrm{A}) p_\mathrm{A}} \underset{N \gg 1}{\approx} \frac{1}{3}\,. \tag{S22}$$

1.3 Both regimes in the neutral case for a cluster size of 1

There is a special case that does not require both assembly regimes to be considered separately: the neutral case ($r_\mathrm{A} = r_\mathrm{B}$) with a cluster size of 1 ($n = 1$). In this case, the probability $\alpha_N^{N_\mathrm{A}}$, given by Equation S2, becomes

$$\alpha_N^{N_\mathrm{A}} = \frac{r N_\mathrm{A} + c p_\mathrm{A}}{r N + c}\,, \tag{S23}$$

while the initial condition of the probability $P(N_\mathrm{A}, N)$, given by Equation S1, satisfies $P(0,0) = 1$. Here again, we can derive the first four moments of N_A. They are given by

$$\langle N_\mathrm{A} \rangle = p_\mathrm{A} N\,, \tag{S24}$$

$$\langle N_\mathrm{A}^2 \rangle \underset{N \gg 1}{\approx} \frac{(c p_\mathrm{A} + r) p_\mathrm{A} N^2}{c + r}\,, \tag{S25}$$

$$\langle N_\mathrm{A}^3 \rangle \underset{N \gg 1}{\approx} \frac{(c p_\mathrm{A} + r)(c p_\mathrm{A} + 2r) p_\mathrm{A} N^3}{(c + r)(c + 2r)}\,, \tag{S26}$$

and

$$\langle N_\mathrm{A}^4 \rangle \underset{N \gg 1}{\approx} \frac{(c p_\mathrm{A} + r)(c p_\mathrm{A} + 2r)(c p_\mathrm{A} + 3r) p_\mathrm{A} N^4}{(c + r)(c + 2r)(c + 3r)}\,. \tag{S27}$$

From the previous moments, we can derive the variance, skewness, and kurtosis, which are respectively given by

$$\sigma^2 \underset{N \gg 1}{\approx} \frac{r(1 - p_\mathrm{A}) p_\mathrm{A} N^2}{c + r}\,, \tag{S28}$$

$$\gamma \underset{N \gg 1}{\approx} \frac{2r(1 - 2p_\mathrm{A})}{(c + 2r)\sqrt{\frac{r(1 - p_\mathrm{A}) p_\mathrm{A}}{c + r}}}\,, \tag{S29}$$

and

$$\kappa \underset{N \gg 1}{\approx} \frac{3(c + r)(2r + (c - 6r)(1 - p_\mathrm{A}) p_\mathrm{A})}{(c + 2r)(c + 3r)(1 - p_\mathrm{A}) p_\mathrm{A}}\,. \tag{S30}$$

Finally, we derive the bimodality coefficient

$$\mathrm{BC} \underset{N \gg 1}{\approx} \frac{(c + 3r)\left(c^2(p_\mathrm{A} - 1)p_\mathrm{A} - 4c(3(p_\mathrm{A} - 1)p_\mathrm{A} + 1)r - 4(3(p_\mathrm{A} - 1)p_\mathrm{A} + 1)r^2\right)}{3(c + r)(c + 2r)((p_\mathrm{A} - 1)p_\mathrm{A}(c - 6r) - 2r)}\,. \tag{S31}$$

2 Extension to S species

In the main text, we consider a microbial pool composed of two species. Here, we generalize the model to include S species, all assumed to replicate at the same rate r. These species are indexed by $i = 1, 2, \ldots, S$ and are present in the microbial pool with respective abundances $p_1, p_2, \cdots, p_S$.

Since the bimodality coefficient is not applicable for microbial communities with more than two species, we quantify diversity using two measures: α-diversity, which captures within-community richness, and β-diversity, which describes between-community dissimilarity.

2.1 α-diversity

2.1.1 Richness

In this work, we define α-diversity as the number of distinct microbial species present in a community when it reaches carrying capacity, i.e., richness. We denote this quantity by α_{R}.

2.1.2 High-dispersal regime

In the limit of high dispersal, the richness of each community is expected to match the full species pool, i.e., $\alpha_{\mathrm{R}} = S$.

2.1.3 Low-dispersal regime

In the low-dispersal regime, community richness corresponds to the number of species present in the initial cluster that disperses from the pool.

Multinomial sampling. The species composition of a cluster of size n is drawn from a multinomial distribution given by

$$P(N_1, N_2, \ldots, N_S) = \frac{n!}{N_1! N_2! \cdots N_S!} p_1^{N_1} p_2^{N_2} \cdots p_S^{N_S}, \tag{S32}$$

where N_i is the number of microbes of species i in the cluster, subject to the constraint

$$\sum_{i=1}^{S} N_i = n, \tag{S33}$$

ensuring the total number of microbes equals the cluster size. Additionally, the abundances in the pool satisfy

$$\sum_{i=1}^{S} p_i = 1. \tag{S34}$$

Clusters with a single species. Consider a cluster composed solely of species i, i.e., all n microbes belong to species i. The probability of such a cluster is

$$P(N_1 = 0, \ldots, N_i = n, \ldots, N_S = 0) = p_i^n. \tag{S35}$$

Summing over all S species, the probability that a cluster contains only one species is

$$P(\alpha_{\mathrm{R}} = 1) = \sum_{i=1}^{S} p_i^n. \tag{S36}$$

Clusters with two species. Next, consider a cluster containing exactly two species, i and j. Such a cluster contains N_i individuals of species i and $n-N_i$ of species j, where $1 \le N_i \le n-1$. The probability for such a cluster is

$$\sum_{k=1}^{n-1} P(N_1=0,...,N_i,...,N_j=n-N_i,...,N_S=0) = \sum_{k=1}^{n-1} \frac{n!}{N_i!(n-N_i)!} p_i^{N_i} p_j^{n-N_i} = (p_i+p_j)^n - p_i^n - p_j^n \,. \tag{S37}$$

Summing over all distinct pairs of species, the probability that a cluster contains exactly two species is

$$P(\alpha_{\mathrm{R}}=2) = \sum_{i=1}^{S-1} \sum_{j=i+1}^{S} \left[(p_i+p_j)^n - p_i^n - p_j^n\right]. \tag{S38}$$

Assuming a uniform pool with $p_i = 1/S$ for all i, this expression simplifies to

$$P(\alpha_{\mathrm{R}}=2) = \frac{1}{2}(2^n-2)(S-1)\left(\frac{1}{S}\right)^{n-1}. \tag{S39}$$

General Case. Following the reasoning above, one can derive a general expression for the probability $P(\alpha_{\mathrm{R}})$ of observing any particular richness level $\alpha_{\mathrm{R}} \in \{1,2,\ldots,n\}$ (see Equation 5 in the main text).

2.2 β-diversity

In addition to quantifying α-diversity, we also assess β-diversity, which measures the dissimilarity between a pair of microbial communities.

2.2.1 Jaccard distance

Here, we focus on the Jaccard distance, a metric that captures the proportion of species that differ between two communities. Mathematically, the Jaccard distance is defined as

$$\beta_{\mathrm{J}} = 1 - J(\mathrm{X},\mathrm{Y})\,, \tag{S40}$$

where $J(\mathrm{X},\mathrm{Y}) = |\mathrm{X}\cap\mathrm{Y}|/|\mathrm{X}\bigcup\mathrm{Y}|$ is the Jaccard similarity coefficient. This coefficient represents the ratio of the number of shared species between communities X and Y to the total number of unique species across both communities. Equivalently, it can be expressed as

$$J(\mathrm{X},\mathrm{Y}) = |\mathrm{X}\cap\mathrm{Y}|/(|\mathrm{X}|+|\mathrm{Y}|-|\mathrm{X}\cap\mathrm{Y}|)\,, \tag{S41}$$

where |X| and |Y| denote the species richness of communities X and Y, respectively. A Jaccard distance of 0 indicates identical species composition, while a value of 1 signifies completely distinct communities.

2.2.2 High-dispersal regime

In the high-dispersal regime, dispersal tends to homogenize community composition, resulting in a Jaccard distance close to zero.

2.2.3 Low-dispersal regime

In contrast, in the low-dispersal regime, the Jaccard distance is influenced by the composition of the initial clusters that populate each community. This case can be modeled by comparing the compositions of two clusters independently sampled from the same multinomial distribution

$$(N_{\mathrm{X},1}, N_{\mathrm{X},2}, ..., N_{\mathrm{X},S}) \sim \mathrm{Multinomial}(n, [p_1, p_2, ..., p_S]) \,, \tag{S42}$$

and

$$(N_{\mathrm{Y},1}, N_{\mathrm{Y},2}, ..., N_{\mathrm{Y},S}) \sim \mathrm{Multinomial}(n, [p_1, p_2, ..., p_S]) \,, \tag{S43}$$

where $N_{\mathrm{X},1}$ is the number of individuals of species 1 in the first cluster that disperses into community X, and similarly for the other variables.

For a given species i, the probability that both clusters contain at least one microbe of that species is

$$q_i = P(N_{\mathrm{X},i} > 0 \;\&\; N_{\mathrm{Y},i} > 0) = (1 - (1 - p_i)^n)^2 \,. \tag{S44}$$

Assuming uniform species abundances, i.e., $p_i = 1/S$ for all i, the number of shared species $|\mathrm{X} \cap \mathrm{Y}| = k$ can be approximated by the following binomial distribution

$$P(k) \approx \binom{S}{k} q^k (1 - q)^{S-k} \,, \tag{S45}$$

where $q = (1 - (1 - 1/S)^n)^2$ is the probability that a given species is present in both clusters.

3 Mean number of clusters contributing to community assembly

Assembly dynamics. We consider the dynamics of a microbial community of size N, constrained by a carrying capacity K. Two types of events contribute to community assembly:

- **Dispersal:** a cluster of size n arrives from the pool at rate

  $$T_{\text{disp}} = c\left(1 - \frac{N}{K}\right),$$

  leading to the update $N \to N + n$.

- **Replication:** a microbe within the community divides at rate

  $$T_{\text{rep}} = rN\left(1 - \frac{N}{K}\right),$$

  corresponding to $N \to N + 1$.

The total event rate is therefore

$$T = T_{\text{disp}} + T_{\text{rep}} = (c + rN)\left(1 - \frac{N}{K}\right).$$

Probability of a dispersal event. Given a community of size N, the probability that the next event is a dispersal is

$$p_{\text{disp}}(N) = \frac{T_{\text{disp}}}{T_{\text{disp}} + T_{\text{rep}}} = \frac{c}{c + rN}. \tag{S46}$$

Importantly, this probability is independent of the saturation factor $1 - N/K$, and thus the result holds for any form of density dependence.

Mean number of clusters contributing to assembly. Each dispersal event increases N by n, whereas replication increases N by 1. Let m_{disp} denote the mean number of dispersal clusters contributing to the community by the time it reaches carrying capacity. Treating N as a continuous variable, the expected increment in m_{disp} per unit increase in N is given by

$$\mathrm{d}m_{\text{disp}} = \frac{p_{\text{disp}}(N)}{\langle \Delta N \rangle}\,\mathrm{d}N, \tag{S47}$$

where the expected change in community size per event is

$$\langle \Delta N \rangle = p_{\text{disp}}(N) \cdot n + \Big(1 - p_{\text{disp}}(N)\Big) \cdot 1 = \frac{cn + rN}{c + rN}. \tag{S48}$$

This yields

$$\mathrm{d}m_{\text{disp}} = \frac{c}{cn + rN}\,\mathrm{d}N. \tag{S49}$$

Integrating from $N = 0$ to $N = K$, the mean number of clusters contributing to assembly is

$$m_{\text{disp}} = \frac{c}{r}\,\ln\!\left(1 + \frac{rK}{cn}\right). \tag{S50}$$

4 Robustness analyses

To ensure that our results are robust to specific modeling assumptions, we perform a series of robustness analyses in which we relax some of the assumptions made in the main model. Specifically, we explored three independent modifications: (i) removing the saturation term from the dispersal rate, so that it is constant, i.e., c, rather than density-dependent, i.e., $c(1 - N/K)$; (ii) introducing a death rate d, kept small relative to the division rate r; and (iii) allowing the cluster size n to vary stochastically by sampling it from a Poisson distribution. Each modification was applied separately to isolate its effect on the results.

4.1 Dispersal without saturation

In the main text, we assumed that saturation affects dispersal in the same way it affects replication, by multiplying the dispersal rate by a logistic term $(1 - N/K)$. This formulation captures scenarios where limited space or other density-dependent constraints reduce both replication and dispersal at high population densities. Here, we relax this assumption and instead consider a model in which saturation does not apply to dispersal, so that the dispersal rate remains constant c regardless of population density.

S7A and S7B Figs show that this modification has no impact on the results: our analytical predictions continue to accurately capture the bimodality coefficient and, thus, the diversity patterns emerging during community assembly. This similarity likely arises because diversity patterns are primarily shaped during the very early stages of community assembly, when $N \ll K$ and thus $1 - N/K \approx 1$, making the saturation term effectively negligible.

4.2 Community assembly with cell death

Next, we extend the model by introducing a death rate, denoted by d. This modification adds two possible events to the Gillespie algorithm:

$$(N_{\mathrm{A}}, N_{\mathrm{B}}) \xrightarrow{dN_{\mathrm{A}}} (N_{\mathrm{A}} - 1, N_{\mathrm{B}}) \quad \text{and} \quad (N_{\mathrm{A}}, N_{\mathrm{B}}) \xrightarrow{dN_{\mathrm{B}}} (N_{\mathrm{A}}, N_{\mathrm{B}} - 1) \, .$$

With death included, the total population no longer stabilizes at K but instead approaches the logistic equilibrium size $K(1-d/r)$, where r is the replication rate, assuming that $r_{\mathrm{A}} = r_{\mathrm{B}} = r$. Accordingly, we stop simulations once this equilibrium is reached.

S7C and S7D Figs show that the results remain very similar to those obtained without death, indicating that our main conclusions are robust to this modification. Note that the death rate d was chosen to be small relative to the replication rate r, ensuring that community extinctions do not occur.

4.3 Random cluster size

In the main text, we assumed that all clusters have the same fixed size. Here, we relax this assumption to test whether our results hold when cluster sizes vary stochastically. To do so, we slightly modify our model implementation so that, at each dispersal event, the cluster size is sampled from a Poisson distribution with mean n. The composition of each cluster is then determined using a binomial distribution, as in the main model.

S7E and S7F Figs show that our results remain qualitatively the same. In the low-dispersal regime, we observe an increase in the bimodality coefficient for cluster sizes larger than one compared to the fixed cluster size case. This arises because the Poisson distribution produces many sampled cluster sizes smaller than the mean, thereby reducing the probability that a cluster contains more than one species, which, in turn, increases the bimodality coefficient.

5 Experimental data analysis

We applied our approach to three datasets collected by [4], who investigated the assembly of the gut microbiota in *C. elegans* by feeding them mixtures of bacteria. The first dataset examines the gut microbiota of AU37 worm strains fed a 50/50 mixture of fluorescently labeled *E. coli* strains (YFP and dsRed) and includes 59 biological replicates (S9A Fig). The second dataset mirrors the first, differing only in the worm strain, which is glp-4, with 24 biological replicates (S9B Fig). The third dataset explores the assembly of the gut microbiota in AU37 worms using a 50/50 mixture of two distinct bacterial species, *S. marcescens* and *E. aerogenes*, also with 24 biological replicates (S9C Fig). Full experimental details are available in [4].

The experimental design of [4] aligns closely with the assumptions of our model. First, they maintained a constant environmental pool in which the relative abundances of the two bacterial strains remained stable over time, mirroring our assumption of a fixed microbial pool composition. Second, they introduced bacteria into initially germ-free hosts: worms were treated with antibiotics to ensure the absence of resident microbes prior to colonization, consistent with our model assumption that communities begin microbe-free. Finally, they used genetically identical (clonal) worms, which supports the assumption that replication rates and carrying capacities are uniform across hosts, as in our framework where all communities share the same parameters.

In their experiment, [4] quantified the structure of worm gut microbiota under different bacterial densities in the environmental pool. Both [4] and [5], who later reanalyzed these data, assumed that increasing bacterial density was equivalent to increasing the dispersal rate, focusing on microbes dispersing individually rather than in clusters, as we do in our study. However, it is also possible that increasing bacterial density leads instead to a rise in cluster size rather than dispersal rate. Importantly, we do not need to distinguish between these two interpretations because Fig 5, S5 Fig, Fig 6, and S6 Fig demonstrate that the bimodality coefficient and mean relative abundance reveal similar patterns of selection, regardless of whether the experimental gradient is driven by changes in dispersal rate or cluster size.

S9 Fig shows the mean relative abundance as a function of the bimodality coefficient for their data sets. As expected, the bimodality coefficient decreases as the bacterial density of the environmental pool, and therefore the dispersal rate or the cluster size, increases, a result already discussed by [4] and [5]. Moreover, from S9A and S9C Figs, it is clear that several bacterial densities give rise to nearly identical bimodality coefficients (BC ≈ 0.8), which corresponds to the plateau observed in the low-dispersal regime (Fig 3A). If the cluster size was equal to one, we would expect to observe more substantial variation in the bimodality coefficient across bacterial densities. The fact that the coefficient instead saturates suggests that clusters larger than one microbe may be present, consistent with Fig 3. However, we acknowledge that our analysis may also be influenced by the relatively low number of worms sampled (24–59 worms), which could make it difficult to reach a bimodality coefficient of 1, since abundance fluctuation distributions built from a limited number of community replicates are inherently noisier.

The data in S9A Fig show that the dsRed-labeled strain is slightly more abundant than the YFP-labeled across bacterial densities and, thus, dispersal rates. This pattern is reminiscent of our model predictions for $p_{\mathrm{A}} > 1/2$ and $s = 0$. Specifically, using the relative abundance averaged over the four data points in S9A Fig, we estimate $p_{\mathrm{dsRed}} \approx 3/5$. The only intrinsic difference between both strains is their fluorescent label, so it is reasonable to assume that they have similar replication rates. The observed asymmetry may therefore reflect differences in the probability of successfully establishing within a host rather than a true imbalance in the microbial pool, which was prepared to be 50/50 [4]. Note that previous studies support this interpretation. [4] found that YFP-labeled bacteria sometimes colonized more slowly than dsRed-labeled bacteria (see their Supplementary Figure S3). [5] quantified relative dispersal

rates ($c_{\text{dsRed}}/c_{\text{YFP}} > 1$), but their model assumes that microbes disperse individually and allow different dispersal rates for each species.

In S9B Fig, the relative abundance of three data points is close to 50%, as their 50% confidence intervals overlap with this value, except for the point corresponding to a bacterial density of 10^9 CFU/mL. This deviation suggests two possible explanations. First, both strains may in fact be neutral in glp-4 worms, and the outlier simply reflects experimental noise, which is plausible given the relatively small sample size (up to 24 worms). Alternatively, the data point may indicate a genuine minimum in mean relative abundance, pointing to a selective disadvantage of the dsRed-labeled bacteria. This hypothesis could be tested by repeating the experiment at higher bacterial densities (e.g., 10^{10} CFU/mL). If the mean relative abundance at this density remains close to 50%, the evidence for a selective disadvantage would be strengthened.

The data in S9C Fig are more challenging to interpret, as they do not follow any clear pattern observed in Figs 5, S4, 6, and S5. Notably, the relative abundance at a bacterial density of 10^6 CFU/mL differs significantly from the other data points. [4] suggested that the low mean relative abundance at low bacterial densities may result from a selective disadvantage of *E. aerogenes* within the worms, whereas the high mean relative abundance at high bacterial densities could reflect the more rapid colonization ability of this species. Although we cannot provide further mechanistic insights, our model predicts that whenever $p_{\text{E}} > 0$ (respectively $p_{\text{E}} < 0$), the mean relative abundance will exceed (respectively fall below) 50%, irrespective of the selection coefficient s (Figs 5, S4, 6, and S5).

References

[1] Loïc Marrec, Claudia Bank, and Thibault Bertrand. Solving the stochastic dynamics of population growth. *Ecology and Evolution*, 13(8), July 2023.

[2] Bahram Houchmandzadeh. Giant fluctuations in logistic growth of two species competing for limited resources. *Physical Review E*, 98(4), October 2018.

[3] Aaron M. Ellison. Effect of seed dimorphism on the density-dependent dynamics of experimental populations of atriplex triangularis (chenopodiaceae). *American Journal of Botany*, 74(8):1280–1288, August 1987.

[4] Nicole M. Vega and Jeff Gore. Stochastic assembly produces heterogeneous communities in the caenorhabditis elegans intestine. *PLOS Biology*, 15(3):e2000633, March 2017.

[5] Loïc Marrec and Claudia Bank. Drivers of diversity within and between microbial communities during stochastic assembly. *Journal of The Royal Society Interface*, 22(232), November 2025.
